# Fractional Fourier Transform-Based Signal Separation for Ultrasonic Guided Wave Inspection of Plates

**DOI:** 10.3390/s24237564

**Published:** 2024-11-27

**Authors:** Chengxiang Peng, Paul Annus, Marek Rist, Raul Land, Madis Ratassepp

**Affiliations:** 1 Department of Civil Engineering and Architecture, Tallinn University of Technology, 19086 Tallinn, Estonia; chengxiang.peng@taltech.ee; 2 Thomas Johann Seebeck Department of Electronics, Tallinn University of Technology, 19086 Tallinn, Estonia

**Keywords:** ultrasonic guided waves, array transducer, signal separation, fractional Fourier transform, time efficiency

## Abstract

Detecting defects in plates is crucial across various industries due to safety risks. While ultrasonic bulk waves offer point-by-point inspections, they are time-consuming and limited in coverage. In contrast, guided waves enable the rapid inspection of larger areas. Array transducers are typically used for more efficient coverage, but conventional excitation methods require sufficient time delays between the excitation of array elements that prolong inspection time, necessitating data acquisition time optimization. Reducing time delays can lead to signal overlapping, complicating signal separation. Conventional frequency domain or time-domain filtering methods often yield unsatisfactory separation results due to the signal overlapping in both domains. This study focuses on the application of the Fractional Fourier Transform (FrFT) for separating overlapping ultrasonic signals, leveraging the FrFT’s ability to distinguish signals that overlap in both the time and frequency domains. Numerical simulations and experiments were conducted to investigate the FrFT’s separation performance for guided waves inspection with array transducers. Results showed that a smaller time delay worsened separation, while using a chirp signal with a broader bandwidth improved separation for signals of fixed duration. Additionally, the effect of signal dispersion on the results was minimal. The findings confirm that the FrFT can effectively separate overlapping signals, enhancing time efficiency in guided wave inspections using array transducers.

## 1. Introduction

Plates are extensively used in various industries, such as construction, aviation, and shipping [1,2,3]. However, defects can form in these metal plates during or after manufacturing. The occurrence of defects can be attributed to various reasons, such as rolling processes or large mechanical impact [4,5]. Different types of defects including holes, cracks, and corrosion are commonly observed in plates [6]. These defects lead to reduced structure integrity, increasing the risk of structural failure, which can, in turn, result in property damage, loss of life, and even catastrophic incidents. For example, the collapse of the I-35W Mississippi River bridge was caused by a defective metal plate, demonstrating that even minor defects can lead to catastrophic failures [7]. Ensuring the proper functioning and safety monitoring of structures incorporating plates is essential, making it crucial to develop reliable methods to detect and quantify these defects.

Numerous techniques have been developed for plate inspection, including radiography, visual inspection, and ultrasonic bulk wave testing. Radiography uses X-rays to penetrate the inspected objects, offering high accuracy and the ability to detect the internal defects beneath the object’s surface. However, X-ray inspection systems pose a risk of radiation exposure to users [8]. Visual inspection performed by humans in industries is simple but not time-efficient and can be tedious, leading to the popularity of camera-based inspection [9]. Camera-based inspection is widely used due to its high accuracy, convenience, and low labor requirements; however, it is generally restricted to surface defects [10]. Ultrasonic bulk wave inspection, while safer than radiography and requiring less complex image processing compared to camera-based methods, involves inspecting objects point by point, which demands high-performance mechanical control systems and is time-consuming [11].

Ultrasonic guided waves (UGWs) have become a promising tool for non-destructive evaluation due to their remarkable advantages. UGW-based inspection methods are cost-effective, capable of detecting both surface and internal defects, and able to inspect large areas without the need to move transducers point by point [12]. Consequently, UGW-based methods are widely employed for both inspecting and long-term monitoring of plates. The use of UGWs in plate inspection has been investigated by numerous researchers. For instance, Cho et al. studied crack growth in plates using UGWs, while Sharma et al. investigated the defects for submerged plates using UGWs [13,14]. UGW inspection techniques can be categorized based on signal reception methods: pulse–echo techniques, which collect reflected signals, and pitch–catch techniques, which collect transmission-through signals [15,16]. Additionally, UGW inspection techniques can be classified according to the number of transducers used: array techniques that utilize multiple transducers and non-array techniques that employ only one or two transducers [17,18].

**Figure 1:**
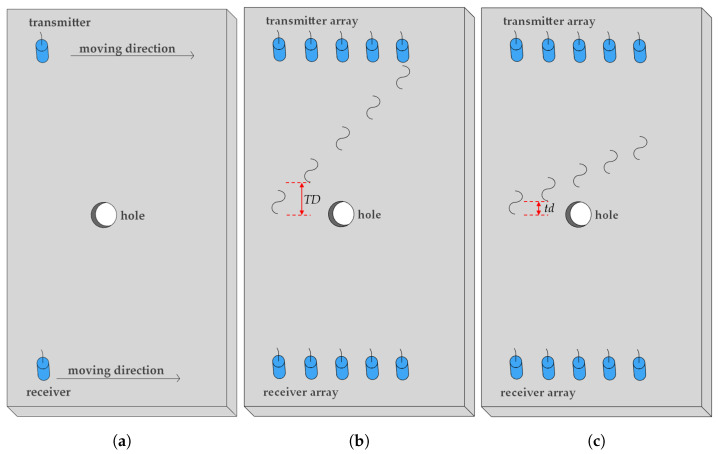
Schematic of UGW inspections. (**a**) Single transmitter–receiver inspection requiring transducer movement for larger areas. (**b**) Array transducer inspection with sufficient time delay. (**c**) Array transducer inspection with reduced time delay.

UGW inspection using a single transducer or a pair of transducers can only scan a limited area, making it inefficient for covering large areas, as shown in Figure 1a. In contrast, an array transducer enables rapid data collection through multiple transducers without needing to move them, facilitating more accurate and efficient inspection over large areas [19]. Conventionally, transducers in an array are excited individually, often with one transducer repeatedly excited multiple times to improve the signal-to-noise ratio through signal averaging [20]. Additionally, a sufficient time delay TD greater than the excitation duration between successive excitations is necessary to ensure that received signals are not interfered with by adjacent excitations, as shown in Figure 1b. However, this approach reduces data acquisition efficiency. To improve the time efficiency of conventional array transducer inspections, the time delay between successive excitations can be shortened to less than the excitation duration, as shown in Figure 1c. By comparing Figure 1b and Figure 1c, the signals emitted by the four sensors on the right in Figure 1c arrive earlier than those in Figure 1b due to the reduced time delay. In conventional array transducer inspection, if the number of signal averaging and transducers in an array is denoted as *P* and *Q*, the total time delay is P×Q×TD. With the reduced time delay td, the total time delay becomes P×Q×td, which can significantly reduce time costs, particularly when the array contains a large number of transducers or requires extensive signal averaging. However, reducing the time delay may cause received signals to overlap, making them challenging to identify within a measured signal. This can complicate signal interpretation, rendering it unsuitable for further data processing.

Various methods have been employed to separate ultrasonic signals. For signals that overlap only in the frequency domain, appropriate frequency filtering can effectively separate them. Similarly, if signals overlap only in the time domain, time-domain windowing can be sufficient for separation. The most challenging case arises when signals overlap in both time and frequency domains, where simple time windowing or frequency filtering alone are inadequate. Eliminating overlap in both domains requires the signal duration to be as short as possible while maintaining a narrow bandwidth. However, according to the uncertainty principle, achieving both a short duration and a narrow bandwidth simultaneously is impossible [21]. Several studies have attempted to address this challenge of dual-domain overlap. High-frequency broadband signals with short durations are often recommended to reduce time-domain overlap, yet high-frequency signals are often impractical due to significant attenuation [22,23]. Frequency-division-based techniques minimize overlap in the frequency domain; however, they often require identical transmitted signals across applications to allow for a consistent interpretation of the received signals, limiting the applicability of frequency-division-based techniques [24,25,26,27]. Matching pursuit methods approximate overlapping signals from a dictionary of atom signals to enable separation in both domains, though constructing an efficient dictionary remains a practical challenge [28,29,30]. Signal coding techniques, such as Barker and Golay codes, help compress signals into narrow peaks, improving signal component localization, but may not always guarantee complete signal separation [31,32].

The limitations of traditional separation methods can be addressed by the Fractional Fourier Transform (FrFT), a generalized Fourier Transform (FT) that has gained increasing attention for ultrasonic signal separation in recent years. Although the concept of the FrFT first appeared in the mathematical literature in the 1920s, it gained significant traction in the 1980s with contributions from Namias [33,34]. Digital computation algorithms for the FrFT, developed by researchers like Ozaktas and Pei have further facilitated its widespread application [35,36,37,38,39,40,41]. Similar to the FT, which decomposes signals into a series of sinusoids, the FrFT represents signals as a decomposition of a series of linear chirps, enabling sharp compression of linear chirp signals. This property allows the FrFT to effectively separate linear chirp signals that overlap in both the time and frequency domains. Recent research has successfully applied the FrFT for ultrasonic signal separation. For instance, Cowell et al. utilized the FrFT to separate echoes generated by a single linear chirp transmitted through a steel plate [42]. Harput et al. explored the FrFT for human tooth imaging, separating overlapping chirp signals for enhanced image clarity [43]. Pachauri applied the short-time FrFT to separate synthetic ultrasonic composite signals [44]. Additionally, Li et al. proposed an FrFT-based method for monitoring pipe elbow erosion by isolating useful signals from interfering signals [45]. Despite these advancements, most FrFT-based ultrasonic signal separation techniques have been limited to single-transducer inspection. Its application in array transducer inspection, along with the effects of chirp parameters and signal dispersion, remain underexplored areas for further research.

The main aim of this paper is to investigate the application of the FrFT in separating overlapping signals for UGW array transducer inspection. To evaluate the effectiveness of the FrFT, we simulated the UGW propagation of the $A_{0}$ mode in an aluminum plate with a hole using finite element (FE) modeling, and conducted corresponding laboratory experiments. Instead of driving each transducer individually, we excited all transducers simultaneously with a reduced time delay to enhance time efficiency. In addition, the performance of the FrFT under different delay settings was compared and analyzed. This paper is organized as follows: Section 2 covers the fundamentals of the FrFT, including its calculation, key properties, and mechanism for separating signals that overlap in both the time and frequency domains. Section 3 validates the separation performance of the FrFT through numerical simulations. Section 4 demonstrates the FrFT’s separation performance based on experimental data. Section 5 discusses the results, and Section 6 provides the conclusions.

## 2. Fractional Fourier Transform Theory and Methods for Signal Separation

### 2.1. Principles of the FrFT

The FrFT has been defined and interpreted in various ways, including as a linear integral transform, a rotation of the time–frequency plane, and as the solutions of differential equations [46,47]. A common definition of the FrFT involves a linear integral transform, expressed as follows:

(1) $$X_{p}\left(u\right)=A_{\alpha }\int_{-\infty }^{+\infty }f\left(t\right)exp\left[j\pi \cot\alpha \left(t^{2}+u^{2}\right)-2ut\csc\alpha \right]dt,$$

where

(2) $$A_{\alpha }=\frac{exp[\frac{-j\pi sgn(\sin\alpha )}{4}+\frac{j\alpha }{2}]}{{\left|\sin\alpha \right|}^{\frac{1}{2}}}$$

and

(3) $$sgn\left(t\right)=\left\{\begin{array}{cc} -1, & t<0\\ 0, & t=0.\\ 1, & t>0 \end{array}\right.$$

Here, f(t) represents the time-domain function to be transformed, *j* is the imaginary unit, and *u* is the fractional domain variable, functioning as the time in a time-domain signal or the frequency of the FT of a signal. The parameter α represents the rotation angle of the FrFT, and the fractional power given by $p=\frac{2\alpha }{\pi }$. The range of α is [−π,+π] with a period of 2π, making *p* range within [−2,2] with a period of 4. The function sgn(t) denotes the sign function.

The chirp signal, characterized by a frequency that varies with time, is widely used in ultrasonic testing due to its frequency-sweeping nature [48]. A common example is the linear chirp signal, where frequency changes linearly over time, defined as

(4) $$c\left(t\right)=exp[2j\pi \left(f_{c}t-\frac{B}{2}t+\frac{B}{2T}t^{2}\right)],0\leq t\leq T,$$

where *T* is the time duration, *B* is the sweeping bandwidth, and $f_{c}$ is the center frequency. The swept frequency range is $\left(f_{c}-\frac{B}{2},f_{c}+\frac{B}{2}\right)$, and the chirp rate is $\frac{B}{T}$. The instantaneous frequency of a linear chirp is defined as

(5) $$f_{i}\left(t\right)=\frac{d}{dt}\left(f_{c}t-\frac{B}{2}t+\frac{B}{2T}t^{2}\right)=f_{c}-\frac{B}{2}+\frac{B}{T}t.$$

By comparing Equations (1) and (4), it is clear that the kernel of the FrFT consists of a set of linear chirps with a chirp rate of cotα, similar to the FT, exp(−2jπft). Unlike the FT, the FrFT includes an additional parameter α, and the FrFT becomes the FT when $\alpha =\frac{pi}{2}$. In the following, $Fr^{\alpha }$ denotes the FrFT with angle α. Some key properties of the FrFT are given as follows [49,50]:

- $Fr^{0}f\left(t\right)=f\left(t\right)$;
- $Fr^{\frac{\pi }{2}}f\left(t\right)$ is equal to the FT of f(t);
- $Fr^{\frac{\pi }{-2}}f\left(t\right)$ is equal to the inverse FT of f(t);
- Linearity: $Fr^{\alpha }\left[\sum_{k=1}^{n}b_{k}f_{k}\left(t\right)\right]=\sum_{k=1}^{n}b_{k}\left\{Fr^{\alpha }\left[f_{k}\left(t\right)\right]\right\}$;
- Index additivity: $Fr^{\alpha +\beta }\left[f\left(t\right)\right]=Fr^{\alpha }\left\{Fr^{\beta }\left[f\left(t\right)\right]\right\}$;
- Rotation invariance: the Wigner–Ville Distribution (WVD) of $Fr^{\alpha }\left[f\left(t\right)\right]$ is identical to the WVD of f(t) with the axis rotated by α.

**Figure 2:**
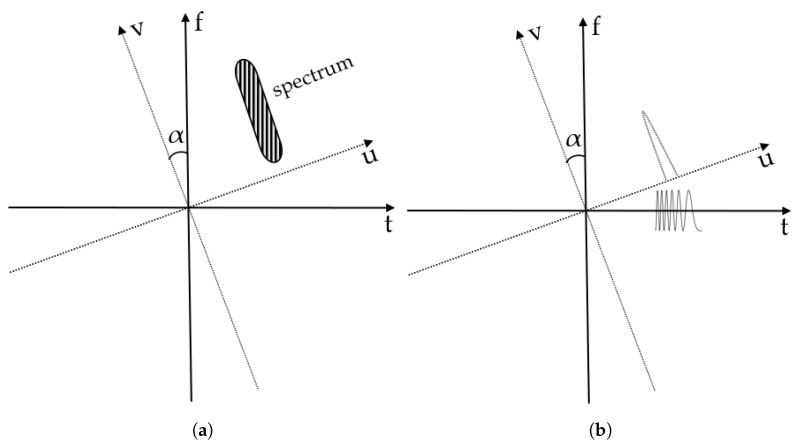
Schematic diagram of the FrFT. (**a**) Rotation invariance: the location of spectral components is unaffected by the rotation angle. (**b**) Chirp compression: the transformation of a long chirp signal into a narrow peak.

The rotation invariance property indicates that the FrFT preserves a signal’s time-frequency distribution. With this, the FrFT can be viewed as a rotation of the time–frequency plane (Figure 2a), where the fractional domain is a domain between the time domain and frequency domain. Additionally, Figure 2b shows that a linear chirp signal can be sharply compressed at an optimal angle in the fractional domain. This optimal angle $\alpha_{o}$ is calculated as $-\arctan(\frac{T}{B})$, with the corresponding fractional power $\frac{-2}{\pi }\arctan\left(\frac{T}{B}\right)$ [51].

### 2.2. Calculation of the FrFT

Similar to how the Fast Fourier Transform (FFT) significantly advanced the development of the FT, the fast calculation algorithm for the FrFT is crucial for its successful application in signal processing. However, unlike the FFT, which strictly adheres to the properties of the FT, current fast digital FrFT calculation algorithms do not fully conform to all FrFT properties of the FrFT [49]. Despite this limitation, some algorithms effectively approximate these properties for practical use. In this study, we employed Ozaktas’ fast FrFT calculation algorithm due to its high accuracy and low calculation complexity [35].

The preliminary step of Ozaktas’ algorithm involves dimension normalization, which equalizes the support for the FrFT regardless of the rotation angle. To achieve this normalization, a scaling factor $S=\sqrt{\frac{\Delta t}{\Delta f}}$ is used to rescale the signal, where Δt and Δf represent the signal’s time length and bandwidth, respectively. Following rescaling, the time and frequency coordinates are transformed into new coordinates as $t_{new}=\frac{t}{S}$ and $f_{new}=fS$, keeping the same signal length $\Delta x=\sqrt{\Delta f\Delta t}$ in both $t_{new}$ and $f_{new}$. The next step is to decompose Equation (1) into simpler equations and perform discretization using Shannon’s interpolation formula. For a digital signal with *N* sampling points, the computational complexity of the algorithm is O(NlogN), which matches that of the FFT. Due to dimension normalization and discretization, the optimal angle $\alpha_{od}$ for the digitized signal, aimed at achieving the best compression of the linear chirp signal c(t) in the fractional domain, is modified as

(6) $$\alpha_{od}=-\arctan\left(\frac{f_{s}^{2}T}{BN}\right),$$

where $f_{s}$ is the sampling frequency.

### 2.3. The FrFT Separation Process for Overlapping Signals

**Figure 3:**
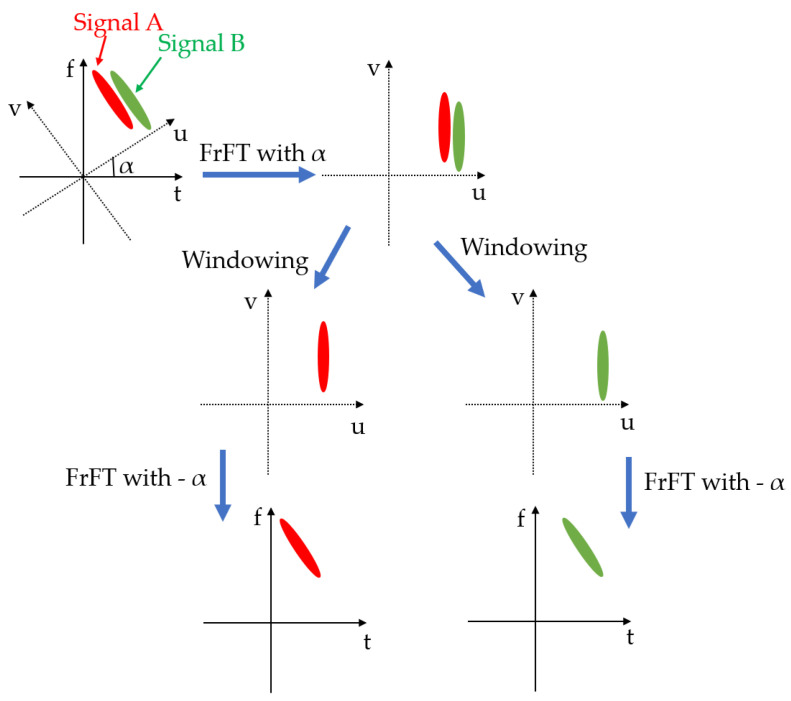
Flow chart of the three-step FrFT separation.

The separation of overlapping linear chirp signals using the FrFT relies on its properties of linearity and rotation invariance. Linearity ensures that the FrFT of a sum of signals equals the sum of the FrFT of each individual signal. Rotation invariance enables the sharp compression of a linear chirp by rotating the axis to a suitable angle. Utilizing these properties, overlapping linear chirp signals in both the time and frequency domains can be decomposed into separated, compressed signals in the fractional domain, as illustrated in Figure 3.

The separation process involves three main steps:

- Transformation to the Fractional Domain: the overlapping signals are transformed into the fractional domain, with the selection of a suitable rotation angle α being critical. Effective separation depends on choosing an optimal angle determined by Equation (6).
- Peak Separation: in the fractional domain, each individual signal appears as a distinct peak. These peaks are separated using a window function, typically a rectangular window, to minimize distortion.
- Restoration to the Original Time Domain: once separated in the fractional domain, the signals are transformed back to the time domain via the inverse FrFT. To achieve signal restoration, the inverse FrFT is employed. Based on the index additivity property, the inverse FrFT can be obtained simply by applying the FrFT with the opposite angle −α, as shown in
  (7) $$f\left(t\right)=Fr^{\alpha -\alpha }\left[f\left(t\right)\right]=Fr^{-\alpha }\left\{Fr^{\alpha }\left[f\left(t\right)\right]\right\}.$$

**Figure 4:**
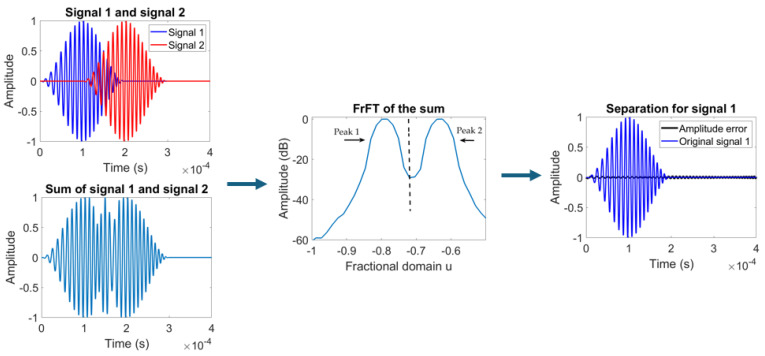
Separation of overlapping linear chirps. The two chirps share a bandwidth of 50 kHz and a center frequency of 100 kHz, with signal 2 delayed by 0.1 ms. Peak 1 corresponds to signal 1, while peak 2 corresponds to signal 2. The boundary between the peaks is marked with the black dashed line. The amplitude error represents the difference between the separated signal and the original signal.

**Figure 5:**
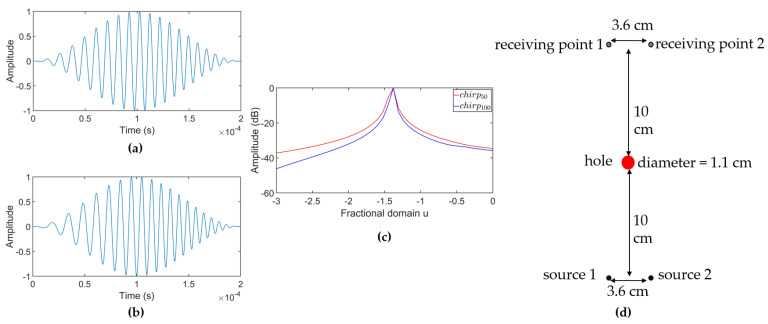
(**a**) $chirp_{50}$. (**b**) $chirp_{100}$. (**c**) The FrFT of $chirp_{100}$ and $chirp_{50}$. (**d**) Geometry of the simulation setup.

Therefore, unlike the inverse FT, the inverse FrFT is simply the FrFT with the opposite angle. A key requirement for separation is a time delay between the overlapping signals, ensuring that the signals are distinguishable in the time–frequency plane even though they overlap in both the time and frequency domains. Figure 4 illustrates the flow of signal separation using the FrFT. Figure 5 provides an example of how a synthetic overlapping signal is separated as two individual signals, where a linear chirp signal is overlapped by another chirp with a time delay.

## 3. Numerical Simulations

### 3.1. Finite Element Modeling

**Figure 6:**
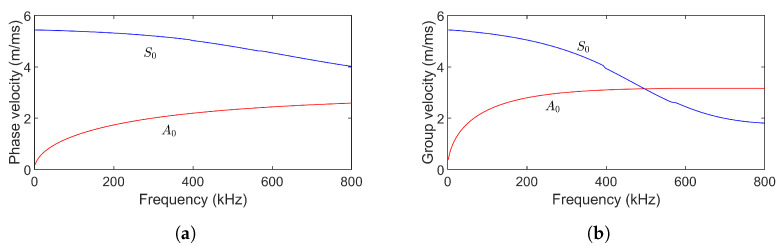
Dispersion curves of Lamb waves in a 2 mm thick aluminum plate. (**a**) Phase velocity. (**b**) Group velocity.

Simulations were performed in ABAQUS software (Version 6.9) [52] to evaluate the FrFT’s effectiveness in separating signals. A three-dimensional (3D) finite element model was created, consisting of an aluminum plate of size 1000 mm × 1000 mm × 2 mm, with an 11 mm diameter hole located centrally. The model was meshed with element type C3D8R with a size of 1 mm. The $A_{0}$ mode was generated by applying an out-of-plane force at two source nodes on the plate surface, while out-of-plane displacements were measured at two receiving nodes. Two different increasing linear chirp signals were used for excitation: the first, $chirp_{50}$ (Figure 5a), is a 20-cycle Hanning-windowed chirp with a 100 kHz center frequency and 50 kHz bandwidth, matching the bandwidth and center frequency of the experimental ultrasonic transducer. The 20-cycle length balances high compression in the fractional domain with minimal plate boundary reflections, as shorter chirps offer less compression, and longer chirps risk interference from boundary reflections. The second signal, $chirp_{100}$ (Figure 5b), retains these parameters but has a 100 kHz bandwidth to evaluate the influence of bandwidth on FrFT performance. The FrFT of $chirp_{100}$ and $chirp_{50}$ is shown in Figure 5c. Dispersion curves for this 2 mm aluminum plate, showing phase and group velocities as functions of frequency, are displayed in Figure 6. Since the excitation signal frequency lies in a dispersive region of the dispersion curve, the signal undergoes shape changes due to dispersion. Both the hole and dispersion effects allow for an assessment of their impact on the FrFT’s separation performance.

Simulations were performed with reduced time delays (see Figure 5d), covering two groups: one with $chirp_{50}$ as the excitation signal and another with $chirp_{100}$. Table 1 lists the time delay settings. Due to the reduced delay, the received signals overlapped in time and were separated using the FrFT.

To assess separation performance, each source was also excited individually, allowing the collection of original, non-overlapping signals for reference. Additionally, simulation 6 used the same delay as simulation 3 but was conducted on a plate without a hole, examining the hole’s impact on separation. To further study dispersion’s influence on separation, two $chirp_{50}$ signals were delayed and summed based on signal locations of simulation 6, and separation was performed on this overlapping summation signal.

### 3.2. Results of Signal Separation

**Figure 7:**
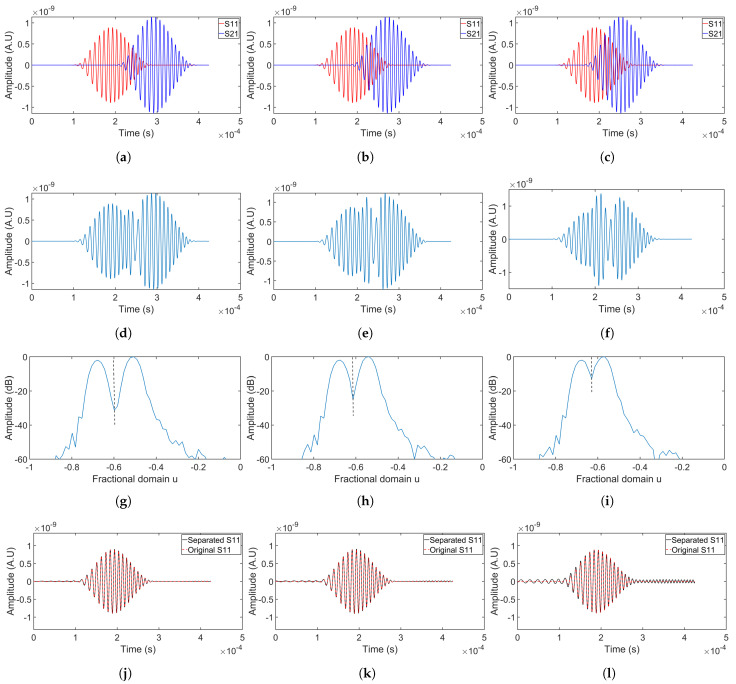

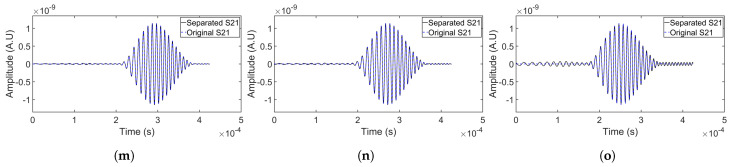
Separation of overlapping signals collected at receiving point 1 using $chirp_{50}$ (simulations 1–3). The first row (**a**–**c**) shows the original signals from each source. The second row (**d**–**f**) represents the summation of these signals, creating overlap. The third row (**g**–**i**) displays the FrFT of the overlapping signals. In the fourth row (**j**–**l**), the separated signal from source 1 is compared with its original signal. The fifth row (**m**–**o**) compares the original signal from source 2 with its separated version.

The signal collected at receiving point 1 when only source 1 is excited was labeled S11, with S21 following the same naming convention. Figure 7 illustrates the detailed separation results for simulation group 1, with each column representing the separation process for simulations 1 to 3 in sequence from left to right. The first-row figures show the original signals from individual sources with different delays. The second row displays the summations of two overlapping signals, showing the complexity of signal interpretation. By applying Equation (6) and using known chirp parameters, the optimal rotation angle was determined. Transforming the signals into the fractional domain using this angle achieved signal compression, as seen in the third-row figures. In this domain, signal components appear as distinct peaks, with smaller delays resulting in greater peak overlap. The peaks were isolated by windowing in the fractional domain, with peak boundaries indicated by black dashed lines. The windowed signals were then transformed to the time domain using the inverse FrFT. The separated signals are compared to the original signals in the fourth and fifth rows, with the fourth row corresponding to S11, and the fifth to S21.

**Figure 8:**
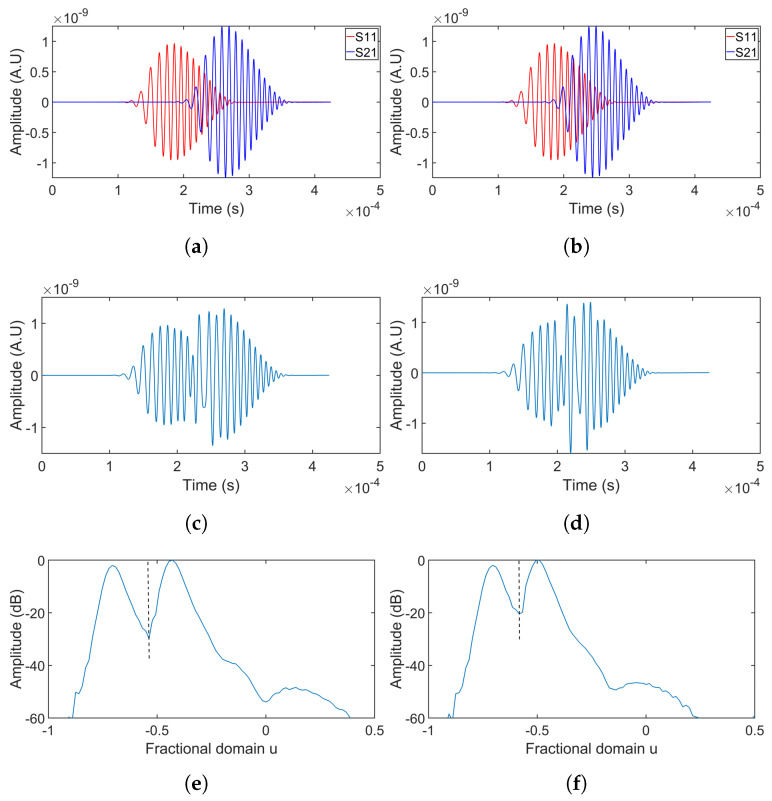

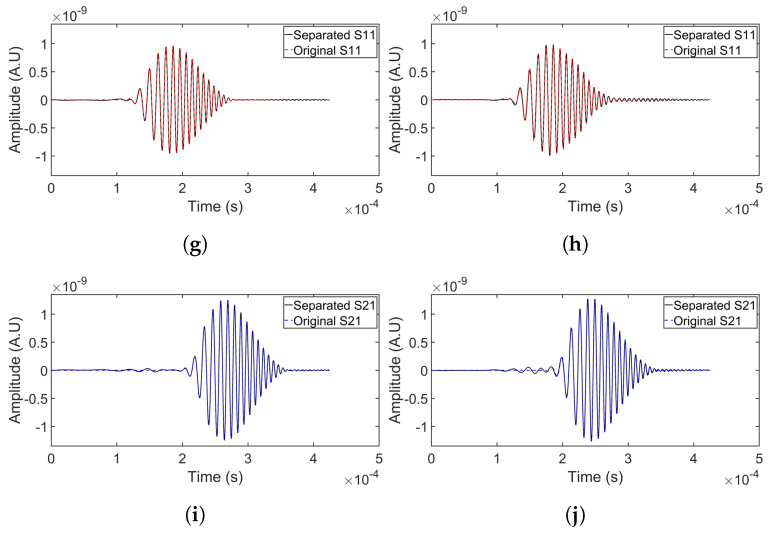
Separation of overlapping signals using $chirp_{100}$ at receiving point 1. The first column shows the separation results for simulation 4, and the results for simulation 5 are shown in the second column. The first row (**a**,**b**) shows the original signals from each source. The second row (**c**,**d**) represents the summation of these signals. The third row (**e**,**f**) displays the FrFT of the overlapping signals. In the fourth row (**g**,**h**), the separated signal from source 1 is compared with its original signal. The fifth row (**i**,**j**) compares the original signal from source 2 with its separated version.

**Figure 9:**
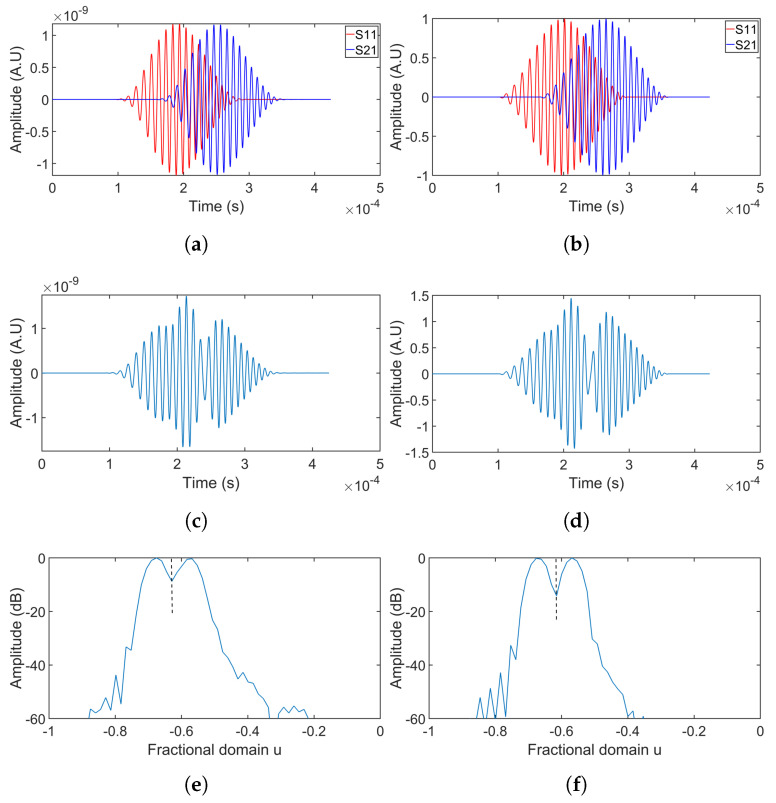

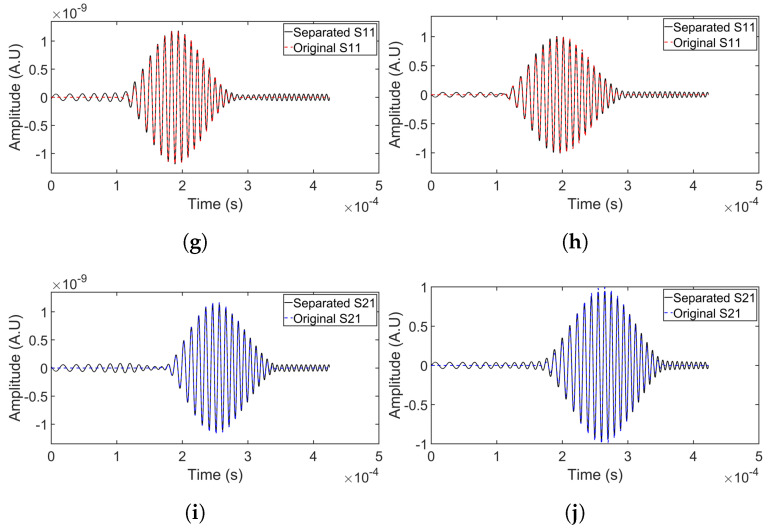
Separation results for simulation 6 and summation of two overlapping $chirp_{50}$. The separation results for simulation 6 are shown in the first column. The separation results for summation of two overlapping $chirp_{50}$ are shown in the second column. The first row (**a**,**b**) shows the original signals from each source. The second row (**c**,**d**) represents the summation of these signals. The third row (**e**,**f**) displays the FrFT of the overlapping signals. In the fourth row (**g**,**h**), the separated signal from source 1 is compared with its original signal. The fifth row (**i**,**j**) compares the original signal from source 2 with its separated version.

Figure 8 presents the separation results for simulations 4 and 5, where $chirp_{100}$ was used. Separation results for simulation 6, which is without a hole, as well as for the summation of two overlapping $chirp_{50}$ without dispersion, are shown in Figure 9.

## 4. Experimental Measurements

### 4.1. Experimental Setup

**Figure 10:**
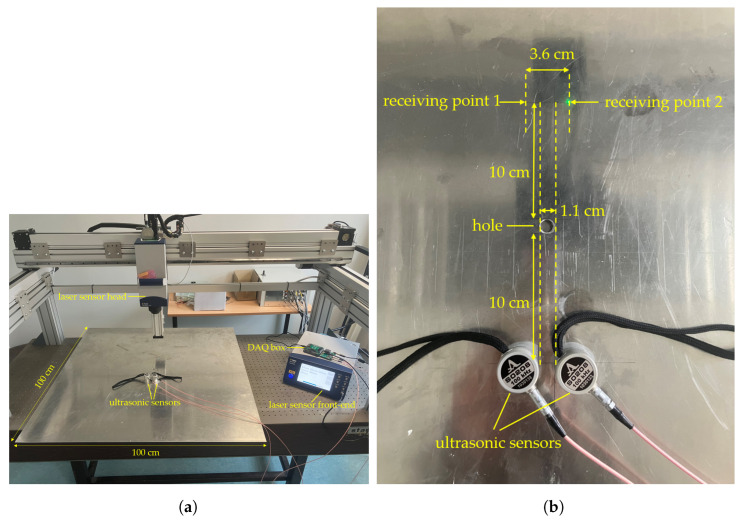
Experimental setup. (**a**) Measurement setup including ultrasonic sensors, laser vibrometer, and DAQ system. (**b**) Geometry of the sensors, hole, and receiving positions.

To further evaluate the FrFT’s effectiveness, experimental measurements were performed to ensure that factors such as transducer response and data acquisition (DAQ) did not significantly impact the separation performance. The experimental setup, shown in Figure 10a, included a 1000 mm × 1000 mm × 2 mm aluminum plate with an 11 mm diameter central hole, two ultrasonic transducers, a laser vibrometer, and a DAQ system. The transducers (S0208, ACS Ltd., Saarbrücken, Germany) with a center frequency of 100 kHz and a bandwidth of 50 kHz were coupled to the plate using a coupling agent for optimal contact. Vibrations at the receiving points on the plate surface were measured using a Polytec VFX-I-160 laser vibrometer. The DAQ box, equipped with functions such as signal amplification and averaging, connected to the PC via USB and was controlled by LabVIEW-based software to set the excitation signals, time delay, sampling rate, amplification, and signal averaging [53].

The experiments focused on $A_{0}$ mode propagation for two excitation sources and two receiving points. The $chirp_{50}$ signal was chosen to validate FrFT separation, while $chirp_{100}$, used in the simulations, was excluded due to the transducers’ bandwidth limitations. Delay settings of experiments 1–3 matched simulations 1–3. The positioning of the hole, transducers, and receiving points in Figure 10b mirrored the simulated setup in Figure 5d. Signal averaging was used in the experiments, with signals collected by the laser vibrometer averaged 100 times to minimize mechanical, ambient, and DAQ system noise.

### 4.2. Results of Signal Separation

**Figure 11:**
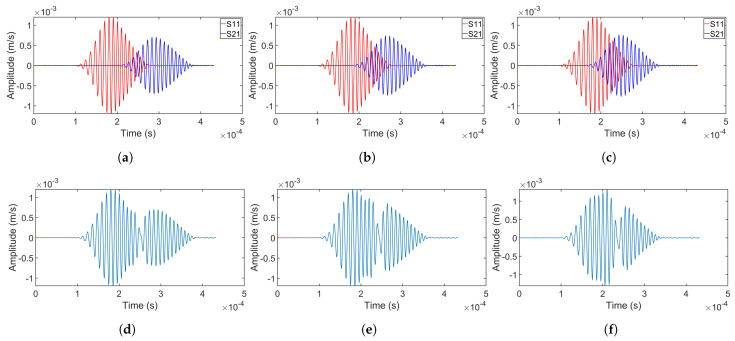

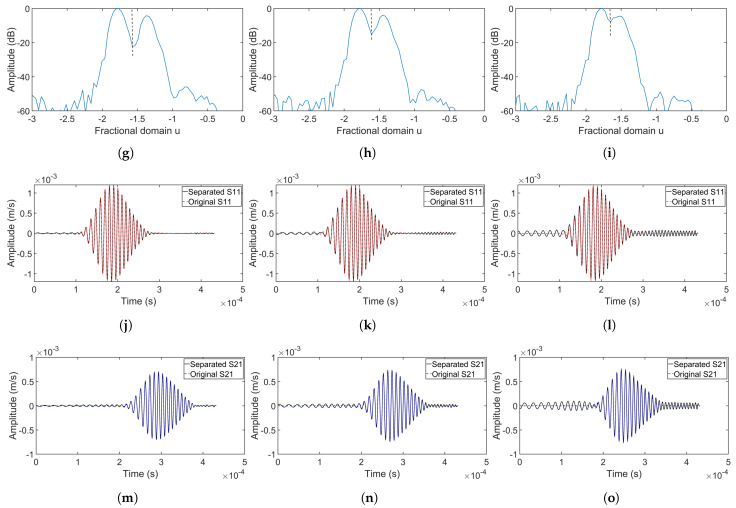
Separation of overlapping signals collected at receiving point 1 (experiments 1–3). The first row (**a**–**c**) displays the original signals from each source. The second row (**d**–**f**) shows the summation of these signals. The third row (**g**–**i**) represents the FrFT of the overlapping signals. In the fourth row (**j**–**l**), the separated signal from source 1 is compared with its original signal. The fifth row (**m**–**o**) compares the original signal from source 2 with its separated version.

Figure 11 illustrates the detailed separation results for experiments, with each column representing the separation process for experiments 1–3 in sequence from left to right. The separation process follows the same flow as in the simulations: the rows from first to fifth correspond to the original signals, the summation of the overlapping signals, the transformed signals in the fractional domain, and the separated signals, respectively.

## 5. Discussions

Observations from simulations 1–3 (Figure 7) indicated that overlap in the fractional domain increased as the time delay decreased, adversely affecting separation performance. The fourth and fifth rows of Figure 7 demonstrated that harmonic-like artifacts at the sidelobes significantly contribute to separation errors. As the delay increased, the peaks in the fractional domain moved further apart, reducing harmonic-like artifacts when applying the rectangular window to separate the overlapping signals. Acceptable separation was achieved for two delays: 100 μs and 80 μs. However, at the 60 μs delay, the separation performance deteriorated rapidly as the difference between the separated signal and the original signal became noticeable at the sidelobes.

Simulations 4–5 (Figure 8) revealed similar trends, confirming that a larger time delay resulted in improved separation performance. Additionally, separation performance using $chirp_{100}$ outperformed that of $chirp_{50}$ under the same time delay.

Figure 9 indicated that the presence of the hole did not significantly impact the FrFT separation performance, as the shape of the signal scattered from the hole was similar to that of the incoming signal. Comparing the dispersive signals shown in the first column with the non-dispersive signals in the figures of the second column, it is evident that the dispersion was mild due to the short wave propagation distance, posing no apparent influence on the separation.

Experiments (Figure 11) also showed that a larger time delay yielded a better separation, and, at the 60 μs delay, the separation performance decreased rapidly. Comparing Figure 7 and Figure 11, it can be observed that experimental measurements demonstrated lower separation accuracy than simulations due to greater separation errors in the sidelobes, particularly at the 60 μs delay.

**Figure 12:**
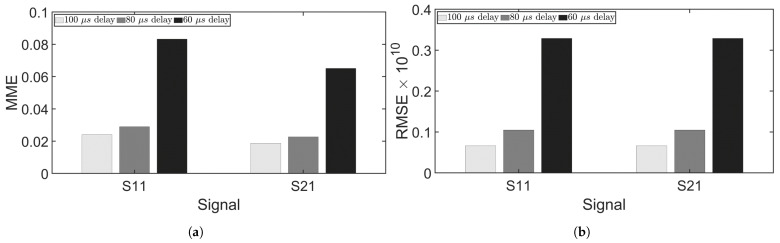
Signal separation errors for simulations 1–3. (**a**) MME. (**b**) RMSE.

**Figure 13:**
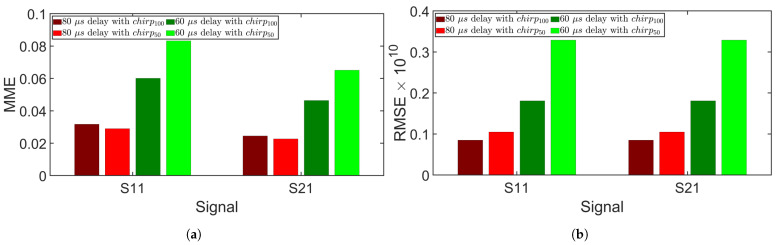
Comparison of signal separation errors using chirp signals with different bandwidths (simulations 4–5). (**a**) MME. (**b**) RMSE.

To quantitatively evaluate the signal separation performance, we employed the maximum magnitude error (MME) and the root mean square error (RMSE) to calculate the error between the original signal and separated signal. MME is defined as the relative magnitude difference among all discrete time points as

(8) $$MME=\frac{\max|S_{o}\left(i\right)-S_{s}\left(i\right)|}{\max|S_{o}\left(i\right)|},$$

where $S_{o}\left(i\right)$ and $S_{s}\left(i\right)$ represent the discrete original and separated signals, respectively. RMSE is defined as the square root of the squared difference between the original signal and separated signal:

(9) $$RMSE=\sqrt{\frac{\sum_{i=1}^{N}{\left[S_{o}\left(i\right)-S_{s}\left(i\right)\right]}^{2}}{N}},$$

where *N* is the number of discrete time points. Figure 12 shows the MME and RMSE for simulations 1–3, revealing that both metrics increased as the delay decreased, indicating that a shorter delay resulted in poorer separation. Figure 13 compares the separation errors between $chirp_{100}$ and $chirp_{50}$. While $chirp_{100}$ exhibited lower MME at the 60 μs delay, it had slightly higher MME at the 80 μs delay. At the 80 μs delay, signals were effectively separated using either $chirp_{100}$ or $chirp_{50}$, with numerical error contributing significantly to the MME. On the other hand, $chirp_{100}$ had smaller RMSE than $chirp_{50}$ at all delays. $chirp_{100}$ favored separation over $chirp_{50}$, which can be intuitively explained by the greater compression of $chirp_{100}$ in the fractional domain. As shown in Figure 5c, the FrFT of $chirp_{50}$ had a larger main lobe width than that of $chirp_{100}$. Figure 12 and Figure 13 collectively demonstrate that smaller time delays lead to greater MME and RMSE. This is due to the increased signal overlap, which becomes a major source of error in FrFT-based signal separation.

**Figure 14:**
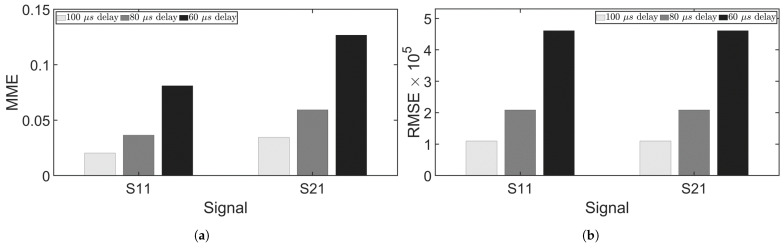
Signal separation errors for experiments. (**a**) MME. (**b**) RMSE.

Experimental measurement errors, depicted in Figure 14, demonstrated that both RMSE and MME increased as the time delay decreased. Notably, separation errors at 60 μs became evident, though the main lobe of the signal remained well separated. By eliminating the signal before and after the main lobe, ideal separation results could still be obtained. It can be observed from Figure 12a and Figure 14a that MME values from experiments were greater than those from simulations. Table 2 lists the MME and RMSE from simulations 1–3 and experiments 1–3 where $chirp_{50}$ was used as the excitation signal. Nonetheless, both the experiments and simulations have shown accurate separation at delays of 100 μs and 80 μs, but accuracy dropped at 60 μs. Overall, the observations from simulations agreed well with the experimental findings, confirming that signal separation can be achieved using the FrFT with an appropriate delay. This method allows for reduced time delays between excitations, facilitating more time-efficient inspections.

## 6. Conclusions

This study presented an efficient method aimed at separating overlapping signals caused by the reduced time delay between excitations, thereby improving the time efficiency of conventional array-transducer-guided wave inspections for plates. The proposed method is based on the FrFT, which enables the separation of signals in the rotated time–frequency domain using an optimal rotation angle. Unlike traditional time or frequency windowing, the FrFT allows for the separation of signals that overlap in both time and frequency domains—a challenge often encountered in practical ultrasonic measurements. The effectiveness of the FrFT was validated through 3D FE simulations and experiments involving varying time delays and bandwidths, demonstrating good agreement between simulation and experimental results. Successful separations were achieved with a minimum time delay of 80 μs, while contamination of the separation process became evident at the 60 μs delay. In addition, using $chirp_{100}$ proved to be more advantageous than $chirp_{50}$ for signal separation. Overall, it was confirmed that larger time delays and wider signal bandwidths enhance separation accuracy, and the FrFT exhibited robustness against dispersion and the presence of holes in simulations.

One limitation of the proposed signal separation method is that it is currently limited to linear chirp signals due to the inherent properties of the FrFT, which restricts its broader application in certain scenarios. Another limitation of this study is the use of only two excitation sources and two receiving points. Future work should explore the application of the FrFT-based separation method with array transducers having a larger number of transducers. Additionally, while the dispersion observed in this study was mild, future research should address scenarios involving severe dispersion. Furthermore, the influence of various types and sizes of defects on the performance of FrFT separation will also be investigated.

## Figures and Tables

**Table 1:** Delay setting for simulations.

Group No.	Simulation No.	Receiving Point No.	Excitation Signal	Source 1	Source 2
1	1	1	$chirp_{50}$	No delay	100 μs delay
	2	1	$chirp_{50}$	No delay	80 μs delay
	3	1	$chirp_{50}$	No delay	60 μs delay
2	4	1	$chirp_{100}$	No delay	80 μs delay
	5	1	$chirp_{100}$	No delay	60 μs delay

**Table 2:** Maximum values of MME and RMSE.

	Sim. No. 1	Sim. No. 2	Sim. No. 3	Exp. No. 1	Exp. No. 2	Exp. No. 3
Time Delay	60 μs	80 μs	100 μs	60 μs	80 μs	100 μs
MME	8.3%	2.9%	2.4%	12.7%	5.9%	3.5%
RMSE	3.3 $\times \;10^{-11}$	1.0 $\times \;10^{-11}$	6.7 $\times \;10^{-12}$	4.6 $\times \;10^{-5}$	2.1 $\times \;10^{-5}$	1.1 $\times \;10^{-5}$

Sim. No.: simulation number. Exp. No.: experiment number.

## Data Availability

The data are available on request from the authors.
